# Fifty Reasons for Being a Homœopath

**Published:** 1888-06

**Authors:** 


### BOOK NOTICES AND REVIEWS.

FIFTY REASONS FOR BEING A HOMŒOPATH. Given by J. Compton Burnett, M. D. London: The Homœopathic Publishing Company, 1888.

It is a real pleasure to read Dr. Burnett's books, for we know of none which better repay one for the reading; they combine instruction with entertainment. We have noticed in the past several of his books which should be read by every one who is skeptical as to the truth of the law of the similars or doubtful as to the efficacy of the potentized drug.

These fifty reasons are given to show an allopath why Homœopathy is worthy an investigation, and they are ample for an unprejudiced person, but, as the author writes to his skeptical friend, the allopath, "Of you personally I have very little hope, for well do I know that though one rose from the dead, yet would you allopaths *not* believe in any, and, therefore, not in my *Fifty Reasons for Being a Homœopalh.*"

This little book ought to be sufficient to persuade any earnest seeker after the truth to put Homœopathy to the one true test, that of bedside experience, and see if it be "found wanting." No other test is worthy the name.

